# Physicochemical and Sensory Properties of Probiotic Fermented Sheep Milk Fortified with Inulin and Sodium Butyrate

**DOI:** 10.3390/molecules31203701

**Published:** 2026-10-15

**Authors:** Katarzyna Szajnar, Małgorzata Pawlos, Julita Drobniak-Łysuniek, Agata Znamirowska-Piotrowska

**Affiliations:** Department of Dairy Technology, Faculty of Technology and Life Sciences, University of Rzeszów, Ćwiklińskiej 2D, 35-601 Rzeszów, Poland; mpawlos@ur.edu.pl (M.P.); jdrobniak@ur.edu.pl (J.D.-Ł.); aznamirowska@ur.edu.pl (A.Z.-P.)

**Keywords:** sheep milk, probiotic bacteria, inulin, sodium butyrate

## Abstract

**Background/Objectives**: In the production of functional foods, bioactive compounds are commonly incorporated into the product, including dietary fibre, polyols, probiotics and prebiotics, unsaturated fatty acids, vitamins, minerals, and other health-promoting constituents. Such ingredients are used, among others, for the fortification of dairy products. From a technological perspective, inulin used in food production is a white powder that dissolves readily in hot water, whereas in cold water it tends to precipitate. It is characterised by a neutral odour and taste. Recent findings, in turn, indicate that sodium butyrate exhibits anti-inflammatory and immunomodulatory effects in intestinal diseases and may be applied in their treatment. The aim of this study was to evaluate the effect of the addition of inulin, as well as inulin combined with sodium butyrate, on the physicochemical characteristics and organoleptic quality of sheep milk fermented with six different probiotic monocultures. **Methods**: Fermented sheep milk samples supplemented with inulin or with the combination of inulin and sodium butyrate were analyzed for instrumental color parameters, pH, titratable acidity, and syneresis. Organoleptic evaluation was also performed. For each probiotic strain, three variants were prepared: a control without additives, a variant with 4% inulin, and a variant with 4% inulin and 0.06% sodium butyrate. **Results**: Supplementation with inulin or with the combination of inulin and sodium butyrate significantly affected instrumental color parameters. In most cases, the pH values of supplemented fermented milk samples were higher than those of the control samples. The highest pH values were observed in milk fermented by *L. rhamnosus* Lr32 and supplemented with inulin or with inulin and sodium butyrate. Moreover, syneresis of milk enriched with inulin as well as with inulin and sodium butyrate was significantly lower than that of the control samples, regardless of the probiotic strain applied. **Conclusions**: Considering the limited availability of probiotic sheep milk products on the market, the obtained results indicate that fermented sheep milk enriched with probiotic strains, inulin, and sodium butyrate may represent a promising matrix for the development of novel functional dairy products. This study also provides new insights into the application of sodium butyrate and inulin in probiotic fermented sheep milk.

## 1. Introduction

In recent years, considerable progress has been observed in research on the development of novel food products containing probiotic bacteria and prebiotics. In the production of functional foods, bioactive compounds are commonly incorporated into the product, including dietary fibre, polyols, probiotics and prebiotics, unsaturated fatty acids, vitamins, minerals, and other health-promoting constituents. Such ingredients are used, among others, for the fortification of dairy products. The noticeable increase in consumer awareness of the functional properties of these products has contributed to the development of this sector of the food industry [1].

The use of prebiotics in dairy products is relatively widespread in many countries. Consumers in the European Union have been familiar with and have consumed prebiotic dairy products for many years. The growing interest in recent years in reduced-calorie products has led to the application of prebiotics in the production of low-fat ice cream, in which sucrose and fat have been replaced with inulin and oligofructose [2]. From a technological perspective, inulin used in food production is a white powder that dissolves readily in hot water, whereas in cold water it tends to precipitate. It is characterized by a neutral odor and taste [3]. The principal technological properties of inulin include its ability to form gels, bind water, form emulsions, and increase viscosity. It improves spreadability, taste, and aroma, and stabilizes products in the form of foams or emulsions [4].

Inulin belongs to the group of inulin-type fructans, which selectively promote the growth of beneficial intestinal microbiota and thereby contribute to host health [5,6,7,8]. Structurally, these compounds consist of fructose units linked by β-(2 → 1) glycosidic bonds, which are resistant to hydrolysis by human digestive enzymes in the upper gastrointestinal tract [9,10]. In the colon, they act as selective substrates for beneficial bacteria, particularly species of the genera *Bifidobacterium* and *Lactobacillus*, which possess β-fructofuranosidase enzymes. Fermentation by these microorganisms leads to the production of short-chain fatty acids (SCFAs) as the main end products. These metabolites exert various physiological effects on the host, providing the basis for many of the health benefits attributed to prebiotics [11]. Following absorption, these metabolites contribute only mini1mally to energy intake, providing approximately 6.3 kJ/g (1.5 kcal/g) [5].

Butyrate is a microbially derived short-chain fatty acid with important physiological functions. However, purified sodium butyrate itself is not classified as a postbiotic according to the current ISAPP consensus definition [12,13]. Recent findings indicate that sodium butyrate exhibits anti-inflammatory and immunomodulatory effects in intestinal diseases and may be applied in their treatment. Sodium butyrate alleviates mucosal inflammation and oxidative stress, restores the impaired epithelial barrier, and modulates visceral sensitivity and intestinal motility. Currently, sodium butyrate (SB), a commonly used form of butyrate, is increasingly applied as an adjunctive agent in the treatment and prevention of various gastrointestinal diseases and disorders, mainly inflammatory diseases, specific or non-specific diarrhoea, functional disorders, dysbiosis-related disturbances, and postoperative conditions, as well as conditions following chemotherapy or radiotherapy [13,14]. Although butyrate has been extensively investigated as a bioactive metabolite and as an additive to starter mixtures, milk, and milk replacers used in animal nutrition, its direct incorporation into foods intended for human consumption, particularly fermented dairy products, remains comparatively underexplored [15,16].

Despite the growing interest in functional dairy products, existing studies have focused mainly on the use of individual ingredients, such as probiotics or prebiotics, whereas their combined application with bioactive metabolites, including sodium butyrate, remains insufficiently explored. There is also limited information on the simultaneous incorporation of inulin and sodium butyrate into fermented sheep milk, as well as on the effects of different probiotic strains on the physicochemical and organoleptic properties of such products. Owing to its distinctive nutritional and physicochemical characteristics, sheep milk represents a promising matrix for the development of functional foods; however, it has received considerably less research attention in this context than cow milk. Therefore, the aim of the present study was to evaluate the effects of different probiotic strains, inulin, and sodium butyrate on the physicochemical and organoleptic properties of fermented sheep milk, thereby expanding knowledge of the potential for developing innovative functional dairy products.

## 2. Results and Discussion

The effects of inulin and the combination of inulin with sodium butyrate on the color parameters of sheep milk fermented by probiotic bacteria are presented in Table 1. 

Fermented sheep milk samples enriched with inulin or with inulin and sodium butyrate and fermented by *L. rhamnosus* Lr32 (GH, GHI), *B. animalis* ssp. *lactis* BB-12 (CD, CDE), and *L. casei* 431 (BC, BCD) were characterized by lower L* values compared with their respective control samples (G, C, and B), indicating a darker color. In contrast, in the remaining experimental variants, the applied supplementation did not significantly affect lightness values (DE, EF, EFG, AB) or resulted in increased lightness of the fermented milk samples (DEF, ABC) relative to the corresponding controls.

All fermented sheep milk samples produced using different probiotic strains exhibited negative a* values, indicating a predominance of the green color component. Supplementation with inulin as well as with inulin and sodium butyrate generally reduced the component of the green color compared with the control samples. An exception was observed for milk fermented by *B. animalis* ssp. *lactis* BB-12, in which the control sample (C) exhibited lower green color intensity compared with CDE and CD samples. Furthermore, analysis of variance (ANOVA) demonstrated that the a* parameter was significantly affected by the bacterial strain used for fermentation and by the interaction between the bacterial strain and the three-level supplementation treatment (Table 4).

In contrast, the b* values determined for samples supplemented with inulin or with inulin and sodium butyrate were generally higher than those recorded for the corresponding control samples, indicating a greater contribution of the yellow color component. The only exceptions were BC, BCD, and ABC samples.

The C* parameter reflects color saturation, with higher values indicating greater color intensity [17]. In most experimental variants, supplementation with inulin or with inulin and sodium butyrate resulted in increased C* values compared with the control samples, indicating more saturated color. Exceptions were observed for BC and ABC samples.

The h° parameter represents the hue angle and describes the perceived color of the analyzed product. In the present study, h° values ranged from 92.84 to 99.96. Furthermore, analysis of variance (ANOVA) showed that the h° parameter was significantly influenced by the bacterial strain used for fermentation, the three-level supplementation treatment, and the interaction between bacterial strain and supplementation treatment (Table 4).

Pimentel et al. [18], in a study investigating the effect of long-chain inulin and *L. paracasei* ssp. *paracasei* on selected properties of yogurt, reported that color parameters were not significantly affected by the addition of either the probiotic strain or the prebiotic. Similarly, Ozturkoglu-Budak et al. [19] observed no significant differences (*p* > 0.05) in L* values between inulin-enriched samples and control samples without inulin supplementation. However, the authors demonstrated that the red–green color parameter (a*) was lower in control samples containing only probiotic cultures compared with synbiotic samples throughout the storage period. Increased a* values in inulin-supplemented samples indicated a shift toward a more reddish color. Moreover, the authors reported that low-degree polymerization (DP) inulin contributed to a greater increase in a* values compared with high-DP inulin or control samples. The b* values obtained in their study were also relatively high, indicating a stronger contribution of the yellow color component than the blue component, which is consistent with the results obtained in the present study.

Changes in the color parameters of fermented milk following inulin supplementation may be related to modifications of the light-scattering properties of the milk matrix. Inulin can interact with milk protein structures and, together with casein micelles and fat globules, influence the way incident light is scattered within the product. Studies using different types of inulin and degrees of polymerization have reported changes in L*, a*, and b* values, although the magnitude and direction of these changes depend on the characteristics and concentration of the inulin and the composition of the dairy matrix [20]. In particular, inulins with higher degrees of polymerization have been associated with slight increases in a* and b* values, which may be related to interactions between inulin and casein micelles. In addition, acidification during fermentation may contribute to structural changes in the casein network and thereby indirectly affect the optical properties of the product [20]. These mechanisms may explain the differences in color observed among the fermented milk variants in the present study.

The pH values of fermented sheep milk were dependent both on the probiotic strain used for fermentation and on the applied supplementation. In most experimental variants, fermented milk enriched with inulin or with the combination of inulin and sodium butyrate exhibited higher pH values than the corresponding control samples. The highest pH values were recorded for GH and GHI samples fermented by *L. rhamnosus* Lr32 and supplemented with inulin or with inulin and sodium butyrate, respectively. In contrast, the lowest pH values were observed in E milk fermented by *L. acidophilus* LA-5 (4.27) (Table 2).

Pereira et al. [21] reported that the addition of inulin to milk fermented by *L. acidophilus* LA-5, *B. lactis* BB-12, and *Streptococcus thermophilus* resulted in significantly higher pH values (*p* < 0.05) compared with probiotic fermented milk without inulin supplementation. Similar observations were obtained in the present study, where milk fermented by *B. animalis* ssp. *lactis* BB-12 and *L. acidophilus* LA-5 supplemented with inulin exhibited higher pH values than the respective control samples. However, different conclusions were reported by Ozturkoglu-Budak et al. [19], who demonstrated that fermented milk samples enriched with inulin were characterized by lower pH values and higher titratable acidity compared with control samples. The authors additionally stated that no significant differences in the pH-lowering effect were observed between low- and high-degree polymerization (DP) inulin in fermented milk produced *using L. acidophilus* LA-5 or *B. animalis* ssp. *lactis* BB-12 cultures.

Table 2 also presents the values of titratable acidity TA (% lactic acid). The determined TA values ranged from 0.70% lactic acid for the GH sample, which simultaneously exhibited the highest pH value, to 1.50% lactic acid for the C sample. Supplementation with either inulin alone or in combination with sodium butyrate resulted in a significant decrease in titratable acidity in samples GH, GHI, DE, CD, CDE, and BC compared with their respective controls. Furthermore, analysis of variance (ANOVA) showed that acidity was significantly influenced by the bacterial strain used for fermentation, the three-level supplementation treatment, and the interaction between bacterial strain and supplementation treatment (Table 4).

The effect of inulin on acidification and pH of fermented milk may be associated with its potential role as an additional substrate for lactic acid bacteria. The availability of fermentable carbohydrates may enhance bacterial metabolic activity and consequently promote organic acid production, resulting in a decrease in pH. Arango et al. [22] reported that the addition of 3.2% inulin increased the acidification rate of yogurt, suggesting that inulin may stimulate the fermentation process under certain conditions. However, the effect of inulin on acidification is not consistent across studies. Guggisberg et al. [23,24] observed no significant effect of inulin addition on the final pH of yogurt, whereas other studies have reported either increased or unchanged acidity depending on the inulin concentration, degree of polymerization, fermentation conditions, and milk composition. Therefore, the effect of inulin on acidification should be considered strain- and formulation-dependent, which may also explain the differences observed among the probiotic strains investigated in the present study.

Syneresis values of fermented sheep milk are presented in Table 2. Samples supplemented with inulin or with inulin and sodium butyrate exhibited significantly lower syneresis than the corresponding control variants, irrespective of the probiotic strain used for fermentation. In selected samples supplemented with inulin and sodium butyrate (GHI, DEF, EFG, and ABC), syneresis was reduced to approximately 1%, which is of considerable technological importance in the production of fermented dairy products. Notably, the control sample E exhibited syneresis of approximately 35%, whereas supplementation with inulin and sodium butyrate reduced this value by more than 33 percentage points. Analysis of variance (ANOVA) showed that syneresis was significantly influenced by the bacterial strain used for fermentation, the three-level supplementation treatment, and the interaction between bacterial strain and supplementation treatment (Table 4).

In addition to its prebiotic properties, inulin exhibits several technological characteristics highly valued in food production, including water-binding capacity [25]. Depending on the degree of polymerization (DP), inulin may be classified as short-chain (DP < 10), medium-chain (DP 2–60), or long-chain (DP > 23) [26,27,28]. Short-chain inulin demonstrates superior water-holding capacity, whereas long-chain inulin is characterized by higher viscosity and improved gel-forming properties, even when applied at relatively low concentrations [27,29]. The reduction in syneresis observed in the inulin-enriched fermented milk may be related to the ability of inulin to modify the water-binding properties and structure of the fermented milk matrix. Inulin can contribute to water retention and affect the development of the protein network during fermentation. Recent research has shown that inulin can participate in the formation of the milk protein network and enhance protein–water interactions, resulting in a more viscous and elastic gel structure with improved structural stability. These changes may increase the ability of the fermented milk matrix to retain water and consequently limit serum separation [29]. In addition, an increase in viscosity associated with inulin incorporation may further restrict the mobility of water within the gel network, thereby reducing syneresis. This interpretation is consistent with the findings of Szymańska et al. [24], who reported a significant reduction in yogurt syneresis with increasing inulin concentration, accompanied by increased viscosity, firmness, elasticity, and overall physical stability. Therefore, the lower syneresis observed in the present study may result from the combined effects of enhanced water retention, modification of protein–water interactions, and development of a more stable and viscous gel network. However, the magnitude of these effects may depend on the concentration and structural characteristics of inulin, as well as on the composition of the milk matrix and fermentation conditions.

Organoleptic evaluation demonstrated that supplementation with inulin as well as with a combination of inulin and sodium (Table 3).

Butyrate significantly affected the consistency of fermented sheep milk. In the GH, GHI, BC, BCD, and ABC samples, the applied supplementation resulted in a less homogeneous consistency that was less characteristic of this type of fermented dairy product compared with the corresponding control samples. In contrast, the panelists rated the consistency of the DE, DEF, EF, EFG, CD, and CDE samples as highly characteristic of this type of fermented dairy product. Furthermore, analysis of variance (ANOVA) showed that consistency was significantly influenced by the bacterial strain used for fermentation and by the interaction between bacterial strain and the three-level supplementation treatment (Table 4).

In most experimental variants, the addition of inulin and the combination of inulin with sodium butyrate intensified the perception of the milky-creamy taste (DE, EF, EFG, CD, CDE, AB, and ABC). The most pronounced sour taste and sour odor were observed in milk samples fermented by *L. johnsonii* LJ, *L. acidophilus* LA-5, and *B. animalis* ssp. *lactis*, both in control and supplemented variants. The results of two-way analysis of variance showed that sour taste and sour aroma were significantly affected by the experimental factors, including the bacterial strain used for fermentation, the three-level supplementation treatment, and the interaction between bacterial strain and supplementation treatment (Table 4).

Importantly, supplementation with inulin as well as with inulin and sodium butyrate generally did not increase the perception of off-taste or off-odor. The only exceptions were EFG and BCD samples supplemented with inulin and sodium butyrate, in which off-taste was evaluated as significantly more perceptible by the sensory panel.

Kip et al. [25] investigated the effect of inulin supplementation on the sensory properties of low-fat yogurts and demonstrated that viscosity, overrun, and density contributed to enhanced creamy mouthfeel, with density being strongly dependent on inulin addition. These observations are consistent with the findings of the present study, in which inulin supplementation intensified the perception of milky-creamy taste.

Our study is a continuation of our previous research on the survival of six probiotic strains during simulated gastrointestinal digestion in fermented sheep milk supplemented with inulin and sodium butyrate [30]. Therefore, the discussion of these results in the present article is highly relevant, and the results from our previous study are appropriately cited [30]. These results showed that the number of viable bacterial cells exceeded 10 log CFU g^−1^ in all analyzed samples. The addition of inulin and sodium butyrate significantly reduced the bacterial cell count only in the sample fermented with *L. rhamnosus* Lr32, compared with the control sample. In contrast, the addition of inulin alone resulted in a significant decrease in the number of viable cells in samples fermented with *L. johnsonii* LJ, *L. acidophilus* LA-5, and *B. animalis* BB-12.

The influence of inulin on the sensory characteristics of fermented milk is mainly related to its effects on texture, viscosity, and mouthfeel. Inulin is widely used as a texture modifier and fat replacer because it can increase viscosity and contribute to a creamier sensory perception. Guggisberg et al. [23] demonstrated that increasing the concentration of inulin in yogurt increased yield stress, firmness, and perceived creaminess, with a strong correlation between instrumental and sensory firmness. Thus, the sensory effects observed in the present study may result from the ability of inulin to modify the rheological properties and microstructure of the fermented milk matrix rather than from its direct contribution to taste.

The effects of sodium butyrate on the physicochemical and sensory characteristics of fermented milk may be associated with both its chemical properties and the characteristic sensory profile of butyrate. From a sensory perspective, butyrate is particularly relevant because butyric acid is associated with a characteristic strong, fatty and rancid-like odor and taste. Recent research using GC-IMS demonstrated that sodium butyrate supplementation can modify the volatile compound profile of milk, including compounds associated with flavor perception [31]. Therefore, the presence of sodium butyrate may contribute to changes in sourness, odor intensity, and overall flavor perception of fermented milk. Its sensory effect may be further modified by fermentation, as the production of lactic acid and other fermentation metabolites can interact with the characteristic sensory profile of butyrate.

## 3. Materials and Methods

### 3.1. Materials

Commercial sheep milk was used to produce fermented milk. The following samples were prepared: a control sample, sheep milk enriched with 4% (*w*/*w*) inulin, and sheep milk enriched with 4% (*w*/*w*) inulin combined with 0.06% (*w*/*w*) sodium butyrate. The fermentation process was carried out using the following probiotic cultures: *Bifidobacterium animalis* ssp. *lactis* BB-12, *Lacticaseibacillus casei* 431, *Lacticaseibacillus paracasei* L26, *Lactobacillus acidophilus* LA-5, *Lacticaseibacillus rhamnosus* Lr-32 and *Lactobacillus johnsonii* LJ Delvo®Pro (Table 5).

### 3.2. Samples Preparation

Chicory-derived inulin (TAR-GROCH-FIL, Filipowice, Poland) was used in the form of a white powder. According to the manufacturer, the product is soluble in water and exhibits gelling properties. The nutritional value per 100 g of product was as follows: energy value, 874 kJ/208 kcal; fat, 0 g, including saturated fatty acids, 0 g; carbohydrates, 7 g, including sugars, 7 g; dietary fiber, 90 g; protein, 0 g; and salt, 0.1 g.

Sodium butyrate (OstroVit, Zambrów, Poland) was used in the form of enteric-coated microcapsules. The microcapsules contained sodium butyrate (600 mg per capsule), providing 180 mg of butyric acid (30%). The coating consisted of fatty acids of plant origin, while hydroxypropyl methylcellulose was used as a bulking agent in the capsule shell.

The obtained samples were stored at 5 °C for 7 days, and on day 7, their physicochemical properties and organoleptic characteristics were evaluated.

### 3.3. Acidity

The pH values of fermented milk samples were determined directly in 100 mL portions using a FiveEasy™ pH meter fitted with an InLab® Solids Pro-ISM electrode (Mettler-Toledo, Greifensee, Switzerland).

Titratable acidity was evaluated according to the ADPI Method #007a [32] with minor modifications. Briefly, 25 g of fermented milk was mixed with 0.5 mL of phenolphthalein indicator and titrated using 0.1 N NaOH until a stable pale pink coloration persisted for 30 s. The results were expressed as % lactic acid (*w*/*v*) and calculated according to the following equation:

$$\mathrm{TA}\;(\%\;\mathrm{lactic}\;\mathrm{acid})\;=\;\frac{NaOH\;titrant,mL\times normality\;of\;NaOH\times 9.008}{sample\;weight,g}$$

### 3.4. Syneresis

Syneresis of fermented milk samples was determined based on the amount of whey separated after centrifugation. A 10 g sample was placed into a 50 mL polypropylene centrifuge tube and centrifuged at 3160× *g* for 10 min at 5 °C using a refrigerated centrifuge (LMC-4200R; Biosan SIA, Rīga, Latvia) [33]. Following centrifugation, the expelled whey was collected and weighed. The obtained values were expressed as the percentage of whey separated relative to the initial sample mass.

### 3.5. Color

Color parameters of fermented milk samples were determined instrumentally using a precision colorimeter (Model NR 145, Shenzhen, China) according to the CIELab color system. Before measurements, the instrument was calibrated against a standard white reference plate [34]. The following color coordinates were recorded: lightness (L*), ranging from 0 (black) to 100 (white), the a* parameter representing the green–red axis, and the b* parameter representing the blue–yellow axis. Based on these values, chroma (C*), indicating color intensity, and hue angle (h°), describing color tone, were additionally calculated.

For the analysis, 100 mL of fermented milk was transferred into a plastic container placed on a white surface. The measuring head of the colorimeter was positioned directly above the sample surface, and the values of L*, a*, b*, C*, and h° were recorded automatically by the instrument.

### 3.6. Organoleptic Evaluation

Organoleptic evaluation of fermented milk samples was performed by a trained panel consisting of 30 assessors (17 women and 13 men aged between 23 and 55 years) in a dedicated sensory analysis laboratory. Prior to evaluation, all participants provided informed consent and were familiarized with the evaluated sensory attributes and the assessment procedure.

The evaluation was conducted in a dedicated sensory analysis laboratory equipped with purpose-designed individual sensory booths. During each session, three coded samples representing the three experimental variants prepared with one probiotic strain were evaluated (approximately 50 mL per sample). Between samples, the assessors rinsed their mouths with still spring water. The interval between the evaluation of consecutive sets of three samples was approximately 30 min. Three sets of three samples were evaluated on the first day, and the remaining three sets of three samples were evaluated on the following day.

A nine-point intensity scale was applied, where 1 corresponded to the lowest intensity or the least characteristic attribute, and 9 corresponded to the highest intensity or the most characteristic attribute. The following attributes were evaluated: consistency, milky-creamy taste, sour taste, sweet taste, off-taste, sour odor, and off-odor [35,36].

The evaluated sensory descriptors were defined according to the methodology of descriptive sensory analysis for fermented milk products [37] as follows:

Consistency—degree of uniformity and texture perception of the product;

Milky-creamy taste—flavor characteristic of milk powder and dairy creaminess;

Sour taste—acidic taste associated with lactic acid;

Sweet taste—taste characteristic of sucrose;

Off-taste—atypical or undesirable taste not characteristic of fermented milk products;

Sour odor—odor associated with lactic acid fermentation;

Off-odor—atypical or undesirable odor not characteristic of fermented milk products.

### 3.7. Statistical Analysis

Statistical analyses were performed using Statistica 13.1 software (StatSoft, Tulsa, OK, USA). The normality of the data distribution was assessed using the Shapiro–Wilk test. As the data met the assumption of normality, parametric statistical methods were applied. The results were expressed as mean values ± standard deviation (SD).

One-way analysis of variance (ANOVA) was performed on the results presented in Table 1, Table 2 and Table 3, considering 18 experimental groups. When significant differences were detected, Tukey’s post hoc test was used to identify differences between individual groups (*p* < 0.05). To evaluate the effects of probiotic strain, three-level supplementation treatment, and the interaction between probiotic strain and supplementation treatment, a two-way analysis of variance was performed. For the analysis of physicochemical properties (pH, titratable acidity, syneresis, and color parameters), a factorial ANOVA model was applied. In contrast, sensory data were analyzed using a repeated-measures model.

For each experimental variant, three independent production batches were prepared. Physicochemical analyses were performed in five technical replicates within each batch, resulting in a total of 15 measurements for each experimental group. Organoleptic evaluation was conducted by 30 trained panelists.

## 4. Conclusions

Considering the limited availability of probiotic sheep milk products on the market, probiotic fermented sheep milk supplemented with inulin alone or in combination with sodium butyrate represents a promising matrix for the development of functional dairy products. Supplementation significantly affected color parameters and generally increased pH compared with control samples, with the highest pH recorded in GH and GHI milk fermented with *Lacticaseibacillus rhamnosus* Lr32. Both supplementation strategies significantly reduced syneresis, regardless of the probiotic strain used. These findings demonstrate the potential of inulin, alone or combined with sodium butyrate, to improve the physicochemical properties of fermented sheep milk.

## Figures and Tables

**Table 1:** Effect of inulin and sodium butyrate on color parameters (L*, a*, b*, C*, h°) of milk fermented by probiotic bacteria.

Experimental Batch	L*	a*	b*	C*	h°
G	95.07 ^d^ ± 0.18	−1.45 ^c^ ± 0.07	10.31 ^b^ ± 0.24	10.41 ^b^ ± 0.25	98.01 ^cd^ ± 0.22
GH	93.83 ^c^ ± 0.47	−1.37 ^d^ ± 0.07	11.17 ^d^ ± 0.54	11.26 ^c^ ± 0.53	97.00 ^c^ ± 0.56
GHI	93.63 ^bc^ ± 0.10	−1.32 ^d^ ± 0.05	11.85 ^de^ ± 0.27	11.92 ^d^ ± 0.28	96.34 ^b^ ± 0.17
D	93.02 ^b^ ± 1.01	−1.53 ^bc^ ± 0.22	10.91 ^c^ ± 1.10	11.02 ^c^ ± 1.07	98.10 ^cd^ ± 1.83
DE	93.04 ^b^ ± 1.44	−1.51 ^bc^ ± 0.23	10.93 ^c^ ± 0.30	11.03 ^c^ ± 0.27	97.91 ^c^ ± 1.35
DEF	95.16 ^d^ ± 0.36	−1.51 ^c^ ± 0.07	11.32 ^d^ ± 0.25	11.42 ^cd^ ± 0.25	97.62 ^c^ ± 0.28
E	92.35 ^b^ ± 1.11	−1.18 ^d^ ± 0.11	10.82 ^c^ ± 0,48	10.89 ^bc^ ± 0.48	96.25 ^b^ ± 0.71
EF	93.61 ^b^ ± 0.21	−0.56 ^e^ ± 0.25	10.99 ^cd^ ± 0.65	11.01 ^c^ ± 0.66	92.84 ^a^ ± 1.20
EFG	93.51 ^b^ ± 0.96	−0.63 ^ef^ ± 0.22	11.32 ^d^ ± 0.33	11.34 ^cd^ ± 0.33	93.17 ^a^ ± 0.99
C	91.27 ^ab^ ± 1.23	−0.82 ^f^ ± 0.27	9.02 ^a^ ± 0.43	9.06 ^a^ ± 0.45	95.15 ^b^ ± 1.48
CD	90.54 ^a^ ± 1.29	−1.39 ^d^ ± 0.03	12.08 ^f^ ± 0.04	12.16 ^e^ ± 0.04	96.57 ^b^ ± 0.14
CDE	90.97 ^a^ ± 1.37	−1.56 ^b^ ± 0.14	12.17 ^f^ ± 0.63	12.27 ^de^ ± 0.64	97.27 ^c^ ± 0.37
B	95.44 ^d^ ± 0.24	−1.63 ^b^ ± 0.16	11.43 ^e^ ± 0.26	11.54 ^cd^ ± 0.27	98.11 ^d^ ± 0.70
BC	94.76 ^c^ ± 0.62	−1.61 ^b^ ± 0.13	10.20 ^b^ ± 0.46	10.32 ^b^ ± 0.47	98.95 ^d^ ± 0.33
BCD	92.87 ^b^ ± 0.26	−1.48 ^c^ ± 0.04	13.03 ^g^ ± 0.17	13.11 ^f^ ± 0.17	96.48 ^b^ ± 0.21
A	94.47 ^c^ ± 0.32	−1.92 ^a^ ± 0.06	10.91 ^c^ ± 0.09	11.08 ^c^ ± 0.10	99.96 ^e^ ± 0.22
AB	94.48 ^c^ ± 1.75	−1.44 ^c^ ± 0.11	11.88 ^e^ ± 0.42	12.03 ^d^ ± 0.51	96.92 ^b^ ± 0.55
ABC	95.36 ^d^ ± 0.46	−1.59 ^bc^ ± 0.06	10.59 ^c^ ± 0.11	10.71 ^b^ ± 0.10	98.56 ^d^ ± 0.32

Mean ± standard deviation; ^a–g^-mean values denoted in columns by different letters differ statistically significantly at *p* ≤ 0.05. A—Control Group with *Lacticaseibacillus paracasei* L26, AB—Group with Inulin 4% with *Lacticaseibacillus paracasei* L26, ABC—Group with Inulin 4% and Sodium Butyrate 0.06% *Lacticaseibacillus paracasei* L26, B—Control Group with *Lacticaseibacillus casei* 431, BC—Group with Inulin 4% with *Lacticaseibacillus casei* 431, BCD—Group with Inulin 4% and Sodium Butyrate 0.06% with *Lacticaseibacillus casei* 431, C—Control Group with *Bifidobacterium animalis* ssp. *lactis* BB-12, CD—Group with Inulin 4% with *Bifidobacterium animalis* ssp. *lactis* BB-12, CDE—Group with Inulin 4% and Sodium Butyrate 0.06% with *Bifidobacterium animalis* ssp. *lactis* BB-12, D—Control Group with *Lactobacillus johnsonii* LJ, DE—Group with Inulin 4% with *Lactobacillus johnsonii* LJ, DEF—Group with Inulin 4% and Sodium Butyrate 0.06% with *Lactobacillus johnsonii* LJ, E—Control Group with *Lactobacillus acidophilus* LA-5, EF—Group with Inulin 4% with *Lactobacillus acidophilus* LA-5, EFG—Group with Inulin 4% and Sodium Butyrate 0.06% with *Lactobacillus acidophilus* LA-5, G—Control Group with *Lacticaseibacillus rhamnosus* Lr32, GH—Group with Inulin 4% with *Lacticaseibacillus rhamnosus* Lr32, GHI—Group with Inulin 4% and Sodium Butyrate 0.06% with *Lacticaseibacillus rhamnosus* Lr32; *p* ≤ 0.05.

**Table 2:** pH value, acidity, and syneresis of milk fermented by probiotic bacteria.

Experimental Batch	pH	TA (% Lactic Acid)	Syneresis %
G	4.96 ^i^ ± 0.05	0.91 ^c^ ± 0.01	18.60 ^g^ ± 2.69
GH	5.23 ^k^ ± 0.02	0.70 ^a^ ± 0.00	8.77 ^e^ ± 0.92
GHI	5.17 ^j^ ± 0.01	0.83 ^b^ ± 0.00	1.00 ^a^ ± 0.00
D	4.43 ^c^ ± 0.01	1.18 ^f^ ± 0.00	14.95 ^ef^ ± 4.14
DE	4.35 ^b^ ± 0.01	1.06 ^d^ ± 0.01	10.69 ^e^ ± 2.66
DEF	4.42 ^c^ ± 0.01	1.31 ^g^ ± 0.02	0.90 ^a^ ± 0.00
E	4.27 ^a^ ± 0.01	1.16 ^e^ ± 0.00	35.04 ^h^ ± 0.39
EF	4.44 ^c^ ± 0.01	1.28 ^g^ ± 0.02	11.22 ^b^ ± 2.94
EFG	4.35 ^b^ ± 0.02	1.31 ^g^ ± 0.01	1.22 ^b^ ± 0.01
C	4.37 ^b^ ± 0.01	1.50 ^h^ ± 0.00	17.45 ^g^ ± 1.16
CD	4.71 ^b^ ± 0.01	1.12 ^e^ ± 0.03	8.34 ^e^ ± 1.15
CDE	4.73 ^h^ ± 0.01	1.18 ^f^ ± 0.01	4.05 ^c^ ± 0.95
B	4.58 ^f^ ± 0.02	1.28 ^g^ ± 0.02	8.37 ^e^ ± 0.22
BC	4.48 ^d^ ± 0.01	1.15 ^e^ ± 0.01	5.93 ^d^ ± 0.71
BCD	4.55 ^e^ ± 0.01	1.27 ^g^ ± 0.04	3.20 ^c^ ± 0.50
A	4.55 ^e^ ± 0.01	1.05 ^d^ ± 0.00	15.03 ^f^ ± 0.68
AB	4.65 ^g^ ± 0.01	1.19 ^f^ ± 0.01	5.05 ^cd^ ± 1.74
ABC	4.56 ^e^ ± 0.02	1.03 ^d^ ± 0.01	1.73 ^b^ ± 0.50

Mean ± standard deviation; ^a–k^-mean values denoted in columns by different letters differ statistically significantly at *p* ≤ 0.05. A—Control Group with *Lacticaseibacillus paracasei* L26, AB—Group with Inulin 4% with *Lacticaseibacillus paracasei* L26, ABC—Group with Inulin 4% and Sodium Butyrate 0.06% *Lacticaseibacillus paracasei* L26, B—Control Group with *Lacticaseibacillus casei* 431, BC—Group with Inulin 4% with *Lacticaseibacillus casei* 431, BCD—Group with Inulin 4% and Sodium Butyrate 0.06% with *Lacticaseibacillus casei* 431, C—Control Group with *Bifidobacterium animalis* ssp. *lactis* BB-12, CD—Group with Inulin 4% with *Bifidobacterium animalis* ssp. *lactis* BB-12, CDE—Group with Inulin 4% and Sodium Butyrate 0.06% with *Bifidobacterium animalis* ssp. *lactis* BB-12, D—Control Group with *Lactobacillus johnsonii* LJ, DE—Group with Inulin 4% with *Lactobacillus johnsonii* LJ, DEF—Group with Inulin 4% and Sodium Butyrate 0.06% with *Lactobacillus johnsonii* LJ, E—Control Group with *Lactobacillus acidophilus* LA-5, EF-Group with Inulin 4% with *Lactobacillus acidophilus* LA-5, EFG (Group with Inulin 4% and Sodium Butyrate 0.06% with *Lactobacillus acidophilus* LA-5, G—Control Group with *Lacticaseibacillus rhamnosus* Lr32, GH—Group with Inulin 4% with *Lacticaseibacillus rhamnosus* Lr32, GHI—Group with Inulin 4% and Sodium Butyrate 0.06% with *Lacticaseibacillus rhamnosus* Lr32; at *p* ≤ 0.05.

**Table 3:** Effect of inulin and sodium butyrate on organoleptic parameters of milk fermented by probiotic bacteria.

Experimental Batch	Consistency	Milky-Creamy Taste	Sour Taste	Sweet Taste	Off-Taste	Sour Odor	Off-Odor
G	4.50 ^b^ ± 1.00	8.75 ^d^ ± 0.50	1.25 ^a^ ± 0.50	6.50 ^cd^ ± 3.00	1.00 ^a^ ± 0.00	2.00 ± 0.00	1.00 ^a^ ± 0.00
GH	2.00 ^a^ ± 1.41	8.25 ^d^ ± 0.96	1.50 ^a^ ± 0.58	7.25 ^d^ ± 1.87	1.00 ^a^ ± 0.00	1.75 ± 0.96	1.00 ^a^ ± 0.00
GHI	3.25 ^a^ ± 0.50	8.00 ^d^ ± 0.20	2.50 ^b^ ± 0.58	7.25 ^d^ ± 1.50	1.00 ^a^ ± 0.00	5.00 ± 0.83	1.00 ^a^ ± 0.00
D	7.00 ^cd^ ± 1.41	6.17 ^c^ ± 1.14	6.67 ^c^ ± 1.21	3.17 ^b^ ± 1.17	1.00 ^a^ ± 0.00	3.50 ± 0.21	1.00 ^a^ ± 0.00
DE	8.00 ^d^ ± 0.89	7.33 ^cd^ ± 1.86	6.50 ^c^ ± 1.38	4.83 ^c^ ± 0.94	1.00 ^a^ ± 0.00	4.00 ± 1.10	1.00 ^a^ ± 0.00
DEF	9.00 ^e^ ± 0.00	4.83 ^b^ ± 1.33	6.50 ^c^ ± 1.76	1.67 ^a^ ± 0.21	1.00 ^a^ ± 0.00	6.17 ± 2.56	1.00 ^a^ ± 0.00
E	3.50 ^a^ ± 0.84	3.00 ^ab^ ± 1.76	7.83 ^c^ ± 1.47	1.83 ^a^ ± 0.41	1.00 ^a^ ± 0.00	5.67 ± 2.16	1.00 ^a^ ± 0.00
EF	6.67 ^c^ ± 1.21	5.17 ^bc^ ± 1.47	6.33 ^c^ ± 1.51	2.50 ^b^ ± 0.22	1.00 ^a^ ± 0.00	3.00 ± 0.63	1.00 ^a^ ± 0.00
EFG	5.50 ^bc^ ± 1.35	5.75 ^bc^ ± 1.50	6.00 ^c^ ± 1.41	4.17 ^bc^ ± 1.47	1.17 ^ab^ ± 0.11	6.20 ± 3.03	1.00 ^a^ ± 0.00
C	5.50 ^bc^ ± 1.00	1.75 ^a^ ± 0.96	7.75 ^cd^ ± 1.89	1.50 ^a^ ± 0.58	1.00 ^a^ ± 0.00	1.75 ± 0.50	1.00 ^a^ ± 0.00
CD	7.50 ^d^ ± 0.58	6.50 ^c^ ± 1.00	3.75 ^b^ ± 1.50	6.00 ^cd^ ± 1.15	1.00 ^a^ ± 0.00	7.00 ± 1.15	1.00 ^a^ ± 0.00
CDE	8.25 ^d^ ± 0.50	3.25 ^ab^ ± 1.89	8.25 ^d^ ± 0.50	2.00 ^ab^ ± 0.41	1.00 ^a^ ± 0.00	7.75 ± 0.96	1.00 ^a^ ± 0.00
B	8.60 ^e^ ± 0.55	4.60 ^b^ ± 1.41	6.00 ^c^ ± 0.71	5.00 ^c^ ± 1.41	1.00 ^a^ ± 0.00	3.20 ± 1.64	1.00 ^a^ ± 0.00
BC	4.20 ^b^ ± 0.84	4.20 ^b^ ± 1.79	3.60 ^b^ ± 0.55	5.00 ^c^ ± 1.03	1.00 ^a^ ± 0.00	2.00 ± 0.71	1.00 ^a^ ± 0.00
BCD	5.40 ^bc^ ± 1.52	5.20 ^bc^ ± 1.28	7.40 ^cd^ ± 1.34	3.60 ^b^ ± 1.82	1.50 ^b^ ± 0.50	6.20 ± 3.27	1.00 ^a^ ± 0.00
A	7.40 ^d^ ± 1.07	6.00 ^c^ ± 1.00	7.60 ^a^ ± 0.89	2.00 ^ab^ ± 0.41	1.00 ^a^ ± 0.00	3.80 ± 1.30	1.00 ^a^ ± 0.00
AB	7.20 ^cd^ ± 1.64	7.00 ^cd^ ± 1.92	3.80 ^b^ ± 2.05	2.20 ^ab^ ± 0.45	1.00 ^a^ ± 0.00	2.80 ± 1.10	1.00 ^a^ ± 0.00
ABC	6.80 ^c^ ± 0.84	6.60 ^c^ ± 1.19	3.60 ^b^ ± 0.89	5.20 ^c^ ± 0.10	1.00 ^a^ ± 0.00	7.80 ± 1.64	1.00 ^a^ ± 0.00

Mean ± standard deviation; ^a–e^-mean values denoted in columns by different letters differ statistically significantly at *p* ≤ 0.05. A—Control Group with *Lacticaseibacillus paracasei* L26, AB—Group with Inulin 4% with *Lacticaseibacillus paracasei* L26,ABC (Group with Inulin 4% and Sodium Butyrate 0.06% *Lacticaseibacillus paracasei* L26, B—Control Group with *Lacticaseibacillus casei* 431, BC—Group with Inulin 4% with *Lacticaseibacillus casei* 431, BCD—Group with Inulin 4% and Sodium Butyrate 0.06% with *Lacticaseibacillus casei* 431, C—Control Group with *Bifidobacterium animalis* ssp. *lactis* BB-12, CD—Group with Inulin 4% with *Bifidobacterium animalis* ssp. *lactis* BB-12, CDE—Group with Inulin 4% and Sodium Butyrate 0.06% with *Bifidobacterium animalis* ssp. *lactis* BB-12, D—Control Group with *Lactobacillus johnsonii* LJ, DE—Group with Inulin 4% with *Lactobacillus johnsonii* LJ, DEF—Group with Inulin 4% and Sodium Butyrate 0.06% with *Lactobacillus johnsonii* LJ, E—Control Group with *Lactobacillus acidophilus* LA-5, EF—Group with Inulin 4% with *Lactobacillus acidophilus* LA-5, EFG (Group with Inulin 4% and Sodium Butyrate 0.06% with *Lactobacillus acidophilus* LA-5, G—Control Group with *Lacticaseibacillus rhamnosus* Lr32, GH—Group with Inulin 4% with *Lacticaseibacillus rhamnosus* Lr32, GHI—Group with Inulin 4% and Sodium Butyrate 0.06% with *Lacticaseibacillus rhamnosus* Lr32; at *p* ≤ 0.05.

**Table 4:** Analysis of variance (ANOVA) *p*-values for the effects of probiotic strain, three-level supplementation treatment, and their interaction on color parameters (L*, a*, b*, C*, h°), pH, titratable acidity (TA), syneresis, and organoleptic attributes (consistency, milky-creamy taste, sour taste, sweet taste, off-taste, sour odor, and off-odor) in milk fermented with probiotic bacteria.

	Probiotic Strain *p*-Values	Three-Level Supplementation Treatment *p*-Values	Probiotic Strain × Three-Level Supplementation Treatment *p*-Values
L*	0.0004 ↑	0.9152	0.5492
a*	*p* < 0.0001 ↑	0.0910	*p* < 0.0001 ↑
b*	0.1661	*p* < 0.0001 ↑	*p* < 0.0001 ↑
C*	0.1207	*p* < 0.0001 ↑	*p* < 0.0001 ↑
h°	*p* < 0.0001 ↑	0.0010 ↑	*p* < 0.0001 ↑
pH	*p* < 0.0001 ↑	*p* < 0.0001 ↑	*p* < 0.0001 ↑
TA (% lactic acid).	*p* < 0.0001 ↑	*p* < 0.0001 ↑	*p* < 0.0001 ↑
Syneresis	*p* < 0.0001 ↑	*p* < 0.0001 ↑	*p* < 0.0001 ↑
Consistency	*p* < 0.0001 ↑	0.3957	*p* < 0.0001 ↑
Milky-creamy taste	*p* < 0.0001 ↑	0.0309 ↑	0.0588
Sour taste	*p* < 0.0001 ↑	*p* < 0.0001 ↑	0.0003 ↑
Sweet taste	*p* < 0.0001 ↑	0.0055 ↑	0.0011 ↑
Off-taste	0.0718	0.1520	0.0726
Sour odor	0.0192 ↑	*p* < 0.0001 ↑	0.0088 ↑
Off-odor	0.1626	0.3426	0.9172

Probiotic Strain × Three-Level Supplementation Treatment = interaction; *p* < 0.05 indicates a statistically significant effect; ↑ = significant interaction.

**Table 5:** Sample Preparation.

Preparation of Milk Samples for Experimental Groups. Milk: I Love My Sheep, Wartberg an der Krems, Austria, per 100 mL 6.0 g Fat, 4.7 g Lactose, 4.6 g Protein, and 0.16 g Salt)					
A, B, C, D, E, G—Sheep milk (control)		AB, BC, CD, DE, EF, GH– Sheep milk + 4% inulin (TAR-GROCH-FIL, Filipowice, Poland)		ABC, BCD, CDE, DEF, EFG, GHI– Sheep milk + 4% inulin (TAR-GROCH-FIL Filipowice, Poland) + 0.06% sodium butyrate (OstroVit Sp. z o.o., (Zambrów, Poland)	
Homogenization (60 °C, 20 MPa)					
Milk Pasteurization (85 °C, 10 min)					
Cooling the Milk (37 °C ± 1 °C)					
Addition of Bacterial Starter Culture					
*Lacticaseibacillus paracasei* L26 (*L. paracasei* L26) Chr. Hansen (Hoersholm, Denmark) A AB ABC	*Lacticaseibacillus casei* 431 (*L. casei* 431) Chr. Hansen (Hoersholm, Denmark) B BC BCD	*Bifidobacterium animalis* *ssp. lactis* BB-12 (*B. animalis* BB-12) (Chr. Hansen (Hoersholm, Denmark) C CD CDE	*Lactobacillus johnsonii* LJ Delvo®Pro (*L. johnsonii* LJ) (DSM, Delft, The Netherlands) D DE DEF	*Lactobacillus acidophilus* LA-5 (*L. acidophilus* LA-5) (Chr. Hansen Hoersholm, Denmark) E EF EFG	*Lacticaseibacillus rhamnosus* Lr32 (*L. rhamnosus* Lr32) (Danisco, DuPont, Copenhagen, Denmark) G GH GHI
Fermentation in an Incubator (37 °C, 10 h)					
Cooling to 5 °C and Storage					
Assessment of Physicochemical and Sensory Properties					

Milk groups obtained in the experiment. *Lacticaseibacillus paracasei* L26 (*L. paracasei* L26): A (Control Group), AB (Gorup with Inulin 4%), ABC (Group with Inulin 4% and Sodium Butyrate 0.06%). *Lacticaseibacillus casei* 431 (*L. casei* 431):B (Control Group), BC (Group with Inullin 4%), BCD (Group with Inulin 4% and Sodium Butyrate 0.06%). *Bifidobacterium animalis* ssp. *lactis* BB-12 (*B. animalis* BB-12): C (Control Group), CD (Group with Inullin 4%), CDE (Group with Inulin 4% and Sodium Butyrate 0.06%). *Lactobacillus johnsonii* LJ (*L. johnsonii* LJ): D (Control Group), DE (Group with Inulin 4%), DEF (Group with Inulin 4% and Sodium Butyrate 0.06%). *Lactobacillus acidophilus* LA-5 (*L. acidophilus* LA-5): E (Control Group), EF (Group with Inulin 4%), EFG (Group with Inulin 4% and Sodium Butyrate 0.06%). *Lacticaseibacillus rhamnosus* Lr32 (*L. rhamnosus* Lr32): G (Control Group), GH (Group with Inulin 4%), GHI (Group with Inulin 4% and Sodium Butyrate 0.06%).

## Data Availability

The original contributions presented in this study are included in the article/Supplementary Material. Further inquiries can be directed to the corresponding author (kszajnar@ur.edu.pl).

## Supplementary Materials

The following supporting information can be downloaded at https://www.mdpi.com/article/10.3390/molecules31203701/s1; Table S1: Viable counts of bacteria (log cfu g^−1^) in milk fermented by probiotic bacteria [31].
