# Supplementary material for: Physicochemical and Sensory Properties of Probiotic Fermented Sheep Milk Fortified with Inulin and Sodium Butyrate
Source: Molecules. 2026 Oct 15;31(20):3701. doi: 10.3390/molecules31203701 (PMC13707777; doi:10.3390/molecules31203701)
Supplement: Supplementary file 1 [file molecules-31-03701-s001.zip › molecules-4571060-supplementary.pdf]

Table S1. Viable counts of bacteria (log cfu g^{-1}) in milk fermented by probiotic bacteria [31].

Probiotic strain	Experimental batch	Log cfu g^{-1}
L. rhamnosus Lr32	Control "G"	11.40^{c}±0.02
	4% inulin "GH"	11.41^{c}±0.31
	4% inulin and 0.06% sodium butyrate "GHI"	10.91^{a}±0.47
L. johnsonii LJ	Control "D"	11.07^{ab}±0.17
	4% inulin "DE"	10.91^{a}±0.06
	4% inulin and 0.06% sodium butyrate "DEF"	10.70^{a}±0.46
L. acidophilus LA-5	Control "E"	10.89^{a}±0.16
	4% inulin "EF"	10.66^{a}±0.40
	4% inulin and 0.06% sodium butyrate "EFG"	11.02^{a}±0.15
B. animalis ssp. *lactis* BB-12	Control "C"	11.19^{b}±0.11
	4% inulin "CD"	11.01^{ab}±0.21
	4% inulin and 0.06% sodium butyrate "CDE"	11.20^{b}±0.10
L. casei 431	Control "B"	11.51^{c}±0.14
	4% inulin "BC"	11.02^{ab}±0.17
	4% inulin and 0.06% sodium butyrate "BCD"	11.36^{c}±0.09
L. paracasei L26	Control "A"	11.26^{b}±0.09
	4% inulin "AB"	11.05^{a}±0.14
	4% inulin and 0.06% sodium butyrate "ABC"	11.09^{ab}±0.11

Mean ± standard deviation; $^{a–h}$—mean values denoted in columns by different letters differ statistically significantly at $p \leq 0.05$" [31].
